# Decline of onset-to-diagnosis interval and its impacts on clinical outcome of COVID-19 in China: a nation-wide observational study

**DOI:** 10.1186/s12879-022-07660-4

**Published:** 2022-08-05

**Authors:** Qing-Bin Lu, Tian-Le Che, Li-Ping Wang, An-Ran Zhang, Xiang Ren, Tao Wang, Meng-Jie Geng, Yi-Fei Wang, Meng-Yang Liu, Hai-Yang Zhang, Li-Qun Fang, Wei Liu, Zhong-Jie Li

**Affiliations:** 1 Department of Laboratorial Science and Technology, School of Public Health, Peking University, Beijing, People’s Republic of China; 2 State Key Laboratory of Pathogen and Biosecurity, Beijing Institute of Microbiology and Epidemiology, 20 Dong-Da Street, Fengtai District, Beijing, 100071 People’s Republic of China; 3 Division of Infectious Disease, Key Laboratory of Surveillance and Early-Warning on Infectious Disease, Chinese Center for Disease Control and Prevention, Beijing, 102206 People’s Republic of China; 4 Department of Epidemiology, School of Public Health, Cheeloo College of Medicine, Shandong University, Jinan, People’s Republic of China; 5 Department of Epidemiology and Health Statistics, School of Public Health, Capital Medical University, Beijing, People’s Republic of China

**Keywords:** COVID-19, Onset-to-diagnosis interval, Severe rate, Case fatality rate

## Abstract

**Background:**

To quantitatively assess the impact of the onset-to-diagnosis interval (ODI) on severity and death for coronavirus disease 2019 (COVID-19) patients.

**Methods:**

This retrospective study was conducted based on the data on COVID-19 cases of China over the age of 40 years reported through China’s National Notifiable Infectious Disease Surveillance System from February 5, 2020 to October 8, 2020. The impacts of ODI on severe rate (SR) and case fatality rate (CFR) were evaluated at individual and population levels, which was further disaggregated by sex, age and geographic origin.

**Results:**

As the rapid decline of ODI from around 40 days in early January to < 3 days in early March, both CFR and SR of COVID-19 largely dropped below 5% in China. After adjusting for age, sex, and region, an effect of ODI on SR was observed with the highest OR of 2.95 (95% CI 2.37‒3.66) at Day 10–11 and attributable fraction (AF) of 29.1% (95% CI 22.2‒36.1%) at Day 8–9. However, little effect of ODI on CFR was observed. Moreover, discrepancy of effect magnitude was found, showing a greater effect from ODI on SR among patients of male sex, younger age, and those cases in Wuhan.

**Conclusion:**

The ODI was significantly associated with the severity of COVID-19, highlighting the importance of timely diagnosis, especially for patients who were confirmed to gain increased benefit from early diagnosis to some extent.

**Supplementary Information:**

The online version contains supplementary material available at 10.1186/s12879-022-07660-4.

## Introduction

Coronavirus disease 2019 (COVID-19) caused by a novel RNA virus (SARS-CoV-2) has widely spread to become the greatest public health challenge to date. By the end of January 2021, over 100 million confirmed cases have been reported across the world, with over 2 million deaths [1], continuing afflicting on the health systems and economy globally. In the majority of cases, COVID-19 is mild, while some developed severe disease, even with fatal outcome in a minority [2]. Case fatality rate (CFR) is one of the key epidemiologic parameters to characterize COVID-19 pandemic, which varied substantially by population demography and across geographic region, and maintained high levels in early stage of the epidemic [3–5]. In the context of countries/regions with continuing epidemic, the estimation of CFR remained challenging, which also hindered a rational estimation of the mortality related factors. Although previous efforts have produced consistent results that older age, male sex as well as underlying comorbidities were risk factors for poor outcomes in COVID-19 cases [6–12], conflicting views remained for many other uncertain factors, such as ethnicity, delayed hospitalization, poverty, obesity, etc. [13–18]. Delay between symptom onset to diagnosis was considered as a risk factor for severe or death outcome [19–24], however, it remained obscure whether these risky behaviors were independently associated with adverse disease, and whether there is modifying effect on the association between them [25].

The epidemic in China has been largely brought under control, as fatal case number since the end of April 2020 has been brought under 5 [26]. As of October 8, 2021, China had reported a total of 85 521 COVID-19 cases [27]. This has allowed a validated analysis for a credible estimation of these undetermined factors using the national reportable data. Here, by performing a nation-wide analysis using a national dataset, we aimed to examine the effect of delay between symptom onset to diagnosis on mortality and severe risk among population subgroups by sex, age, and epidemic regions.

## Methods

### Data sources

Since COVID-19 was included in the list of Chinese notifiable infectious disease on January 21, 2020, each COVID-19 case was required to report to China CDC though the National Notifiable Infectious Diseases Surveillance System of China (NNIDSS) [28]. Individual data, including demographical information, exposure history, clinical manifestations and signs, physical examinations [e.g., chest X-ray or computed tomography (CT) scanning], clinical complications, and underlying condition were collected from the medical records.

### Patient eligibility criteria

This retrospective study was performed based on the data of confirmed COVID-19 cases reported through NNIDSS from February 5, 2020 to October 8, 2020. According to the National Health Commission of the People's Republic of China “Diagnosis and Treatment Program for Novel Coronavirus Infected Pneumonia” [29], all COVID-19 cases were confirmed by real-time polymerase chain reaction (PCR) and clinical manifestations. In addition, in the early stage of the epidemic, medical imaging examinations such as CT and X-rays were also used to confirm cases in Wuhan. We excluded people younger than 40 years old due to their low mortality and low severe rates. In addition, asymptomatic cases and imported cases from abroad were also excluded from the study.

### Outcomes

This study used death and clinical severity as main study outcomes. Severe and mild cases were classified by the “Diagnosis and Treatment Scheme of New Coronavirus Infected Pneumonia” [29].

### Ethics declaration

National Health Commission of the People’s Republic of China decided to collect and analyze the COVID-19 case data to control the outbreak. This study was approved by the ethical review committee of the Chinese Center for Disease Control and Prevention (2020-026). Data were de-identified, and informed consent was waived by the ethical review committee of the Chinese Center for Disease Control and Prevention.

### Statistical analysis

For confirmed patients with COVID-19, the onset-to-diagnosis interval (ODI) was defined as the interval in days from symptom onset to laboratory diagnosis. In China, patients with positive nucleic acid tests were defined as confirmed cases after they had clinical symptoms or signs, so the ODI of all cases was greater than or equal to 0.

The median and interquartile range (IQR) were used to describe the ODIs, which were compared among sex, and age groups (40‒59 years, 60‒69 years, and ≥ 70 years) by Wilcoxon rank-sum test or Kruskal–Wallis rank-sum test. The ODI, severe rate (SR) and CFR in all the cases were delineated and compared between two regions (Wuhan vs. outside Wuhan).

The factors potentially associated with ODI were analyzed using multivariate linear regression model, which had accounted for sex, age and region. The profile of SR and CFR in response to the ODI was explored by Join-Point regression (JPR) model to identify the turning points of ODIs at which the significant change of SR and CFR could be marked. Given that CFRs and SRs followed abnormal distribution, natural log-linear model was used to estimate the linear trends of annual percentage change (APC) as recommended. Under a log-linear model, rates change at a constant percentage by each day of the interval, was calculated as a fixed APC. Positive or negative value of APC with statistical significance indicated an increasing or decreasing trend respectively, while non-significant (P ≥ 0.05) APC was considered as stable trend.

Logistic regression model was applied to evaluate the effects of ODI on the severe or fatal outcome. For the categorical grouping, the ODI within 19 days was subgrouped by a two-day interval, i.e., 0‒1, 2‒3, 4‒5…, and 18‒19. A series of logistic regression models were applied to evaluate the impact of different ODIs with the shortest interval of 0–1 day used as reference. All analysis was performed with sex, age and geographic region included, based on which the adjusted odds ratio (OR) and 95% confidence interval (CI) were estimated. In addition, attributable fraction (AF) of ODI to severe or fatal outcome was estimated by age, sex and region.

We estimated the possible overall numbers of severe cases and deaths under predesigned 16 scenarios, in which if all patients were diagnosed within 0, 1, 2, …, and ≥ 15 days after symptom onset. Based on the real SRs and CFRs reported under different ODIs from 0 to ≥ 15 days in 12 patient subgroups classified by age groups (40‒59 years, 60‒69 years, and ≥ 70 years), sex (male and female), and regions (Wuhan and outside Wuhan), we projected the numbers of severe or deceased cases for each subgroups and respectively for each of the predesigned 16 scenarios, and then the overall number of severe or deceased cases were estimated by summating the numbers of 12 subgroups for each of 16 scenarios.

All data analyses were performed using the R 3.6.2 (R Foundation for Statistical Computing, Vienna, Austria). All *P*‐values were two sided and a P‐value < 0.05 was considered as significant.

## Results

**Fig. 1:**
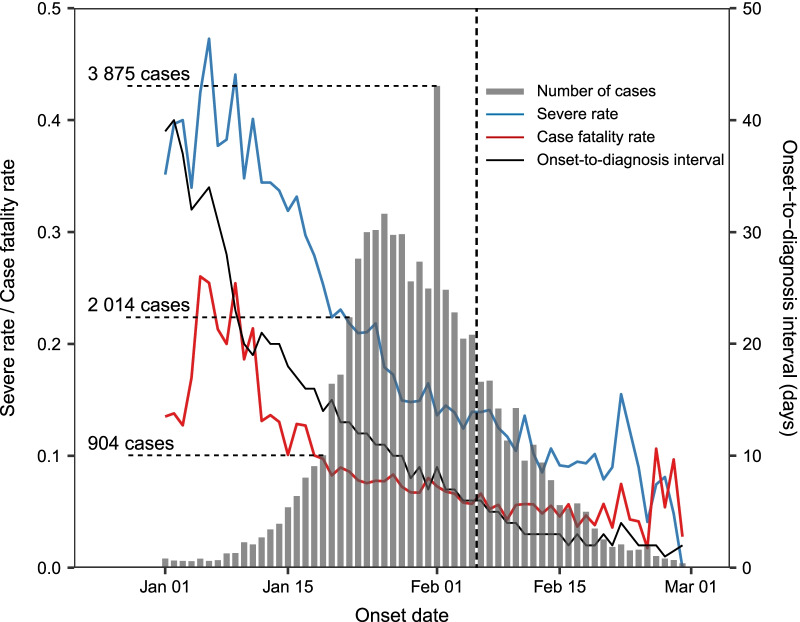
The temporal pattern of cases number, severe rate, case fatality rate, days in ODI for all COVID-19 patients in the mainland of China from January to March 2020. The left vertical axis corresponded to the daily severe rate and case fatality rate; the right vertical axis corresponded to the ODI. Cases after March, 2020 are not shown due to the small proportion

From 5 February 2020 to 8 October 2020, a total of 16 077 COVID-19 cases over the age of 40 years old (47.5% male, 21.8% aged ≥ 70 years, 71.7% from Wuhan) were recruited in 31 provinces in the mainland of China (Table 1). Severe and fatal outcomes were recorded from 1 792 and 837 cases, giving a SR of 11.1% and CFR of 5.2%, respectively. Compared with other regions in China, Wuhan had a higher proportion of patients aged ≥ 70 years (23.6% vs. 17.4%), as well as significantly higher rates of severe outcome and deaths. As the rapid decline of ODI from about 40 days in early January 2020 to < 3 days in early March, both COVID-19 related CFR and SR largely decreased, till to below 5% (Fig. 1). The ODI, as well as the SR and CFR were maintained at a similar level among most provinces of China during the entire epidemic period except Wuhan (Additional file 1: Fig. S1).

**Table 1:** The demographical characteristics and onset-to-diagnosis intervals (ODI) of COVID-19 cases

Variables	All cases			Wuhan			Outside Wuhan			P*
	n (%)	ODI, M (IQR)	*P*	n (%)	ODI, M (IQR)	*P*	n (%)	ODI, M (IQR)	*P*	
Sex			< 0.001			< 0.001			0.158	
Male	7 640 (47.5)	4 (2–7)		5 307 (46.1)	4 (2–7)		2 333 (51.2)	3 (1–5)		< 0.001
Female	8 437 (52.5)	4 (2–7)		6 216 (53.9)	5 (2–8)		2 221 (48.8)	3 (1–5)		< 0.001
Age, years			< 0.001			0.433			< 0.001	
40–59	8 456 (52.6)	4 (2–7)		5 669 (49.2)	4 (2–8)		2 787 (61.2)	3 (1–5)		< 0.001
60–69	4 114 (25.6)	4 (2–7)		3 139 (27.2)	5 (2–8)		975 (21.4)	3 (1–6)		< 0.001
≥ 70	3 507 (21.8)	4 (2–7)		2 715 (23.6)	4 (2–8)		792 (17.4)	3 (1–5)		< 0.001
Severe			< 0.001			< 0.001			0.002	
No	14 285 (88.9)	4 (2–7)		10 112 (87.8)	4 (2–8)		4 173 (91.6)	3 (1–5)		< 0.001
Yes	1 792 (11.1)	5 (2–9)		1 411 (12.2)	6 (3–9)		381 (8.4)	3 (2–6)		< 0.001
Death			0.971			0.047			0.655	
No	15 240 (94.8)	4 (2–7)		10 794 (93.7)	4 (2–8)		4 446 (97.6)	3 (1–5)		< 0.001
Yes	837 (5.2)	4 (2–7)		729 (6.3)	4 (2–7)		108 (2.4)	3 (1–5)		< 0.001
Total	16 077 (100.0)	4 (2–7)		11 523 (100.0)	4 (2–8)		4 554 (100.0)	3 (1–5)		

*Comparison on the medians of ODI between Wuhan and Outside Wuhan. *M* median; *IQR* interquartile range; *COVID-19* coronavirus disease 2019

### ODI and related factors

We profiled the ODIs in terms of age and sex for Wuhan and outside Wuhan separately. The overall median ODI was 4 days (IQR 2‒7), while Wuhan had longer median ODIs than outside Wuhan (Table 1, Additional file 1: Fig. S2). In general, the ODI appeared to be slightly longer in female cases and cases aged 60‒69 years, and notably longer in Wuhan and among both severe cases than their counterparts (all *P* < 0.001, Table 1). However, fatal cases did not appear to have a longer ODI relative to non-death cases.

Multivariate linear regression model further revealed significantly longer ODIs in female, cases aged 60‒69 years and cases in Wuhan (Additional file 1: Table S1). Subgroup analysis showed that the pattern of a longer ODI in sex was consistently seen in both regions, but cases aged 60‒69 years did not have a longer ODI in Wuhan.

### Profile of SR in relate to ODI

**Fig. 2:**
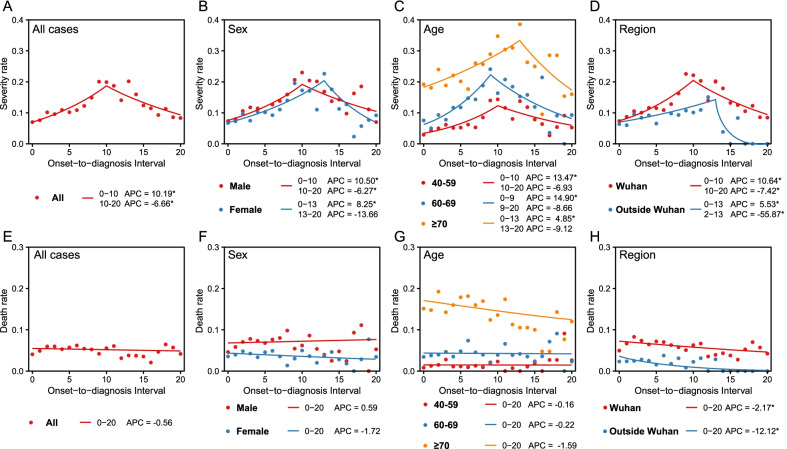
The patterns of ODI-related COVID-19 disease severe rate and case fatality rate examined by Join-Point regression models. **A**‒**D** indicate the overall severe rate and that stratified by sex, age and regions, respectively. **E**–**H** indicate the overall case fatality rate and that stratified by sex, age and regions, respectively. For each panel, red and blue points indicate severe rates and case fatality rates at each day of ODI, which were fitted by the red or blue curve. The arrows indicate the turning points of fitted curves. The Annual Percent Change (APC) value of each fitted curve was provided for each panel. *APC is significantly from zero at alpha = 0.05 level

As a whole the SR increased from 7.0% to 20.0% and then decreased to 8.0%, corresponding to the increase of ODI from 0 to 20 days, with a turning point observed at Day 10 of the ODI. Before the turning point, the APC of SR was 8.61% (*P* < 0.05), but after that it was -6.66% (*P* < 0.05) (Fig. 2A).

With the increase of ODI, earlier appearing of turning points and higher APCs of SR were found in patients of males over females (10 days *vs.* 13 days for the turning points, and 10.50% *vs.* 8.25% for APCs), in patients aged 40‒59 years, 60‒69 years, and aged ≥ 70 years (10 days, 9 days and 13 days for the turning points, 13.47%, 14.90% and 4.85% for APCs), and outside Wuhan over Wuhan (10 days *vs.* 13 days for the turning points, 10.64% *vs.* 5.53% for APCs), respectively (Fig. 2B‒D). Given the same ODI, older age and from Wuhan both led to a higher SR (Fig. 2C‒D). The turning points of ODI for those aged < 70 years were significantly longer outside Wuhan than in Wuhan, while it was comparable and those aged ≥ 70 years between two regions (Additional file 1: Fig. S3).

### Impact of ODI on severity of patients with COVID-19

**Fig. 3:**
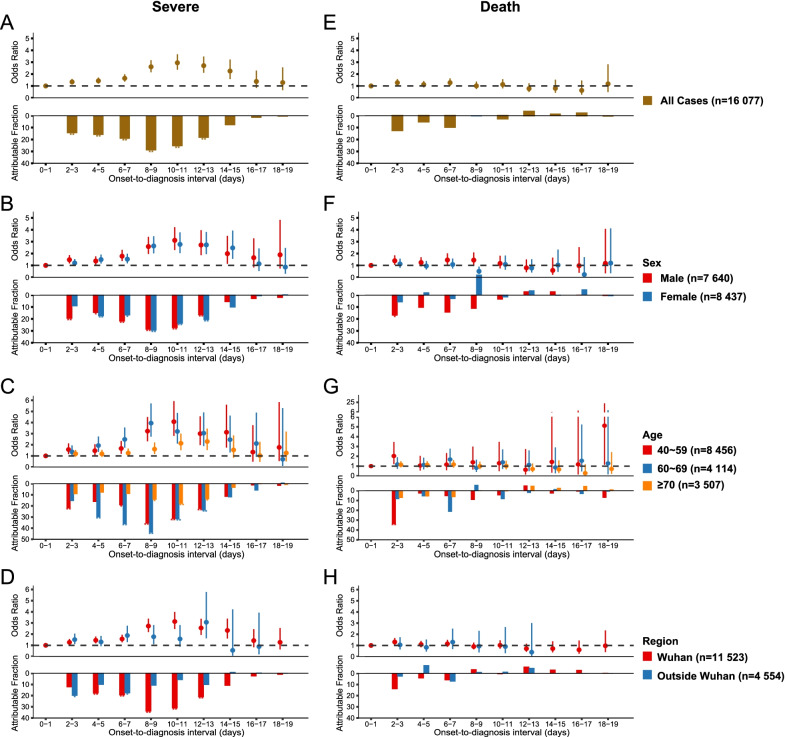
The odds ratio and attributable fraction of COVID-19 disease severe rate and case fatality rate (CFR) from ODIs in the mainland of China. **A** Severe rate for all cases. **B** Severe rate stratified by sex. **C** Severe rate stratified by age. **D** Severe rate stratified by region. **E** Case fatality rate for all cases. **F** Case fatality rate stratified by sex. **G** Case fatality rate stratified by age. **H** Case fatality rate stratified by region. The points and lines represent odds ratios and their 95% CIs. The bars represent the attributable fractions and their significance of differences by asterisk (*P < 0.05; **P < 0.01)

A serial of SR-related ORs and AFs were evaluated based on two-day grouping of ODIs, and also separately delineated by sex, age and region over the whole study period (Fig. 3A‒D, Additional file 1: Tables S2‒S3). All-data based ODI risk functions showed a monotonic pattern, with the highest OR of 2.95 (95% CI 2.37‒3.66) at Day 10–11 and AF of 29.1% (95% CI 22.2%‒36.1%) for severe outcome of COVID-19 cases observed at Day 8–9 after adjusting for age, sex and region (Fig. 3A). A similar pattern was observed for the sex- or age-specific ORs and AFs of severe outcome, disclosing more robust adverse effects of ODI for younger cases (Fig. 3B‒3C). The effect of ODIs on SR was comparable between two regions until to prolonged ODI of Day 8‒9 (Fig. 3D). The magnitude of the association between ODI and severe disease was continuously elevated in Wuhan, with the highest OR and AF observed at Day 10–11 and Day 8–9, while the OR and AF were elevated to the highest level at Day 12–13 and Day 2–3 outside Wuhan. Lower ORs of ODI for severe outcome in cases aged ≥ 70 years were observed both in Wuhan and outside Wuhan (Additional file 1: Fig. S4).

### Profile of CFR in relate to ODI

There appeared to be only a slight decrease in CFR as ODI increased from 0 to 20 days (*P* > 0.05) (Fig. 2E). In addition to a slight increase in CFR in male cases, a slight decrease in CFR was also observed in all age and gender subgroups, with increasing ODI (Fig. 2F‒G). However, the linear correlation between CFR and ODI was significant both in Wuhan and outside Wuhan, with APCs of − 2.17 and − 12.12, respectively (Fig. 2H). When two regions were considered respectively, only female cases and cases aged 60‒69 years outside Wuhan had a significant positive association between CFR and ODI, with APCs of 6.99% and 7.88%, respectively (Additional file 1: Fig. S3).

### Impact of ODI on CFR of COVID-19

A serial of CFR-related ORs and AFs were evaluated based on two-day grouping of ODIs, and were also separately delineated by sex, age and region over the whole study period (Fig. 3E‒H, Additional file 1: Table S4‒S5). Although ODIs of 2‒3 days were shown to have significant impacts on death outcome (*P* < 0.05), the magnitude of this risky effect was minor, with OR of 2.17 and AFs from 12.4%. Significant differences were observed for sex-, age-, and region-specific ORs and AFs of death outcome, disclosing the adverse effects of ODI on death outcome were only seen among male cases, younger cases aged 40‒59 years, and cases from Wuhan (Fig. 3F‒H). Notably, male cases displayed an increasing risk of COVID-19 death as the increase of ODI, with the highest OR of 1.39 (95% CI 1.05–1.85) and AF of 16.9% (95% CI 0.8–33.1%) at the ODIs of Day 2–3, while no such effect was observed for female cases (Fig. 3F, Additional file 1: Table S4‒S5). An increased risk of death from the increase of ODI was observed from patients aged 40–59 years, with the OR of 2.03 (95% CI 1.20–3.46) and AF of 33.6% (95% CI 9.0–58.2%) at the ODIs of Day 2–3, while no such effect was observed for other age groups (Fig. 3G, Additional file 1: Table S4‒S5). The effect of ODI on fatal outcome was only observed in Wuhan, with the OR of 1.31 (95% CI 1.03–1.67) and AF of 13.9% (95% CI − 0.3 to 28.2%) at the ODIs of Day 2–3, while no such effect was observed outside Wuhan (Fig. 3H, Additional file 1: Tables S4‒S5). Among the cases in Wuhan, higher ORs of ODI for fatal outcome were demonstrated in male than in female, in the 40–59 years group than in the ≥ 60 years group (Additional file 1: Fig. S5). Among the cases outside Wuhan, insignificant associations were observed in most two-day groups of ODI stratified by sex and age.

### Prediction of overall numbers of severe cases and deaths for different scenarios

**Fig. 4:**
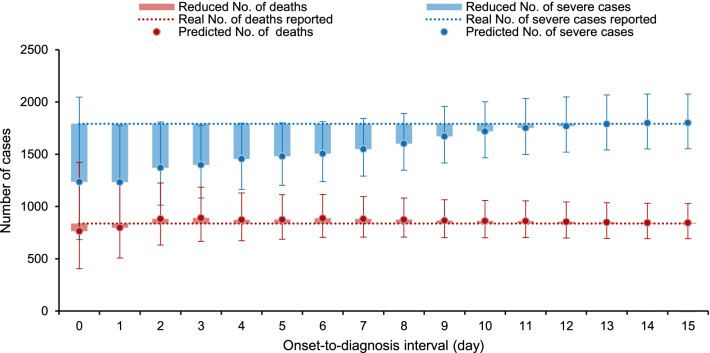
The predictive and real numbers of severe and death cases according to the different cutoff value of ODI

The estimated overall numbers of severe cases ranged from 1 236 (95% CI 683‒2 047) to 1 801 (95% CI 1 553‒2 076) under the 16 scenarios, which were significantly increasing with the prolonged ODI. If all cases were diagnosed within 10 days after symptom onset, the predicted severe cases were estimated to be 1.4 times of the scenario when all cases were diagnosed with 0 or 1 day after symptom onset (Fig. 4 and Additional file 1: Table S6). If all cases were diagnosed within 15 days after symptom onset, the predicted number of severe cases was further increased to 1 801 (95% CI 1 553‒2 076). For prediction of deaths in different scenarios, the estimated overall numbers ranged from 764 (95% CI 405‒1 422) to 844 (95% CI 692‒1 029), which slightly increased in relate to the prolonged ODI, and with much lower magnitude than those of severe cases. If all cases were diagnosed before 3 days after symptom onset, only little reduction of death cases was obtained, which was close to the actual number of death cases reported.

## Discussion

There were previous studies that have determined the association between delayed hospitalization and adverse outcome, however, yielding inconsistent results owing to the discrepancy in terms of the considered outcome, the sample size or the epidemic regions [19–24]. Herein by performing an integrated data analysis on the reported cases in China, we provided first evidence that early diagnosis contributed to the decrease of COVID-19 SR, however the impact on CFR was little. Moreover, a complicated interaction was observed, indicating a greater effect from ODI on SR among patients of male sex, aged 60‒69 years, and those cases in Wuhan. From the perspective of public health, early diagnosis contributed to the causal relationship from patients with younger age on the severe outcome, in that it had taken account of over 1.7‒35.4% in the group of the 40‒59 years and − 0.8 to 43.8% in the group of the 60‒69 years related severe outcome. However, only exerting minor effect on the causal relationship was observed for the patients aged age ≥ 70 years.

Moreover, the diverging patterns in the effects of ODI observed between men and women remained stable across geographic regions, supporting that the ODI related reduced SR observed between sex and among age were free of geographic interference. These findings would favor the view that the vulnerability to ODI differed among the population, which might be the result of a differential host response to the disease. The underlying physiological mechanisms by which ODI trigger differential COVID-19 mortality in sex and age was postulated to be mediated by age and sex specific immunological responses, since for such an emerging and high contagious disease as COVID-19, no heterogenicity on medical measures might be responsible for the apparent age or sex differences.

Aging had been well known to lead to numerous physiological changes, including the deterioration of the immune system, rendering the elderly more susceptible to infections. Senescence of the immune system in the elderly led to increased levels of tissue and circulating proinflammatory cytokines in the absence of an immunological threat where accumulating dysfunctional subsets contribute to immune failure [30–32]. Age-related changes in host immune activity in response to COVID-19 disease had been identified in previous study which involved alterations in the composition and functional declines of diverse immune cells [33]. During process of disease progression, the effect from ODI was highly likely to be overwhelmed by the predominant role of aging, as the age increased, thus a greater effect was observed from the younger group instead.

The sex difference in eliciting differential host response against SARS-COV-2 infection has been studied. According to Takahashi et al.’s study, a more robust T cell response was observed among female patients than male patients at baseline, and poor T cell responses were associated with future progression of disease in male patients [34], which was related to a more rapid disease progression in male than in female patients. Therefore, male might be more vulnerable to longer ODI in progressing into severe outcome, due to the host immune response against SARS-COV-2 inherent to male. These differences in the baseline immune capabilities in male and female patients during the early phase of SARS-CoV-2 infection provide a potential basis for sex-dependent effect from ODI.

After the COVID-19 break, the Chinese government sent a large number of medical staff to Wuhan from all over the country and established several temporary hospitals, which greatly relieved the pressure on the medical system in Wuhan [35, 36]. However, due to the still large number of COVID-19 cases, the accessibility of medical services in Wuhan is still lower than that in other parts of China, which leads to a longer ODI for COVID-19 patients in Wuhan. In addition, poor access to medical services also made patients with severe illness not being able to seek medical treatment in time in Wuhan, while patients with longer ODI in other regions may be due to milder symptoms. Therefore, we suggest that the case classification mechanism should be improved to prioritize hospitalization for patients who are more likely to develop severe disease when a pandemic arrives.

There are some limitations in our study. First, we cannot distinguish the effects of the factors on the disease outcome of COVID-19, such as availability of medical resource, types of treatment, health condition of patients and so on. In many studies, the general reduction in disease mortality and risk along the epidemic progress has been associated with medical care policy, such as improvements in diagnosis capacity available, health-care systems (e.g., rapid hospital admission and improved treatment of patients) [37, 38], reduction in risk factors (e.g., the intervention policy of social distancing, isolation and quarantine), and planned policies led by governments and public health agencies [39–43]. All these policies had been reflected by a rapid diminishing of the diagnosis delay which was witnessed after the major measured were adopted. Second, changes in case definitions and intensity of testing along with the epidemic might make a difference in the detection rate of patients with different severity, and could influence the estimates for the effects of ODI on the SR and CFR. We have rechecked the clinical records of patients to include patients using a standard diagnosis criterion, thus could minimizing the bias from misclassification.

In summary, we determined the correlation between prolonged ODI and increased CFR and higher SR, with a stronger correlation observed for male and younger patients aged < 70 years old, which might be explained by the age-specific and sex-specific differences in host immunity responses to COVID-19. The reduction in risk of mortality that differed regarding age and sex highlights the importance of taking a sex- and age-based approach to the overall treatment and management, especially when considering sex differences in the immune response to SARS-COV-2 infection. The current finding may also change the health-seeking behavior of patients who were confirmed to gain increased benefit from early diagnosis to some extent.

## Supplementary Information

**Additional file 1: Fig S1.** Geographical distributions of onset-to-diagnosis interval, severe rate (SR) and case fatality rate (CFR). **Fig S2.** Frequency distribution of onset-to-diagnosis interval of confirmed COVID-19 cases by regions and epidemic periods. Fig S3: The onset-to-diagnosis interval-related COVID-19 disease severe rate and case fatality rate stratified by regions. **Fig S4.** The odds ratio and attributable fraction of COVID-19 disease severe rate resulted from onset-to-diagnosis intervals in Wuhan and outside Wuhan. **Fig S5.** The odds ratio and attributable fraction of COVID-19 case fatality rate resulted from onset-to-diagnosis intervals in Wuhan and outside Wuhan. **Table S1.** The subgroup analysis on the onset-to-diagnosis interval of patients with COVID-19 for age, sex, geographic region and epidemic period by multivariate linear regression. **Table S2.** Odds ratio of onset-to-diagnosis interval for the risk of severe COVID-19 by multivariate logistic regression model.  **Table S3.** Attributable fraction of onset-to-diagnosis interval for the risk of severe COVID-19. **Table S4.** Odds ratio of onset-to-diagnosis interval for the risk of fatal COVID-19 by multivariate logistic regression model. **Table S5.** Attributable fraction of onset-to-diagnosis interval for the risk of fatal COVID-19. **Table S6.** The predicted and actual case number of severe and death cases according to the different cutoff value of onset-to-diagnosis interval.

## Data Availability

The datasets used and/or analysed during the current study are available from the corresponding author on reasonable request.

## Abbreviations

AF: Attributable fraction
CFR: Case fatality rate
ODI: Onset-to-diagnosis interval
OR: Odds ratio
SR: Severe rate

## References

[1] World Health Organization. Coronavirus disease (COVID-2019) situation dashboard. 2020. https://covid19.who.int/ (2020). accessed 20 Feb 2022.

[2] Wang D, Hu B, Hu C (2020). Clinical characteristics of 138 hospitalized patients with 2019 novel coronavirus-infected pneumonia in Wuhan, China. JAMA.

[3] Drefahl S, Wallace M, Mussino E (2020). A population-based cohort study of socio-demographic risk factors for COVID-19 deaths in Sweden. Nat Commun.

[4] Hauser A, Counotte MJ, Margossian CC (2020). Estimation of SARS-CoV-2 mortality during the early stages of an epidemic: a modeling study in Hubei, China, and six regions in Europe. PLoS Med.

[5] Rentsch CT, Kidwai-Khan F, Tate JP (2020). Patterns of COVID-19 testing and mortality by race and ethnicity among United States veterans: a nationwide cohort study. PLoS Med.

[6] Channappanavar R, Perlman S (2020). Age-related susceptibility to coronavirus infections: role of impaired and dysregulated host immunity. J Clin Invest.

[7] Grasselli G, Greco M, Zanella A (2020). Risk factors associated with mortality among patients with COVID-19 in intensive care units in Lombardy, Italy. JAMA Intern Med.

[8] Gupta S, Hayek SS, Wang W (2020). Factors associated with death in critically ill patients with coronavirus disease 2019 in the US. JAMA Intern Med.

[9] Lu QB, Jiang WL, Zhang X (2020). Comorbidities for fatal outcome among the COVID-19 patients: a hospital-based case–control study. J Infect.

[10] Onder G, Rezza G, Brusaferro S (2020). Case-fatality rate and characteristics of patients dying in relation to COVID-19 in Italy. JAMA.

[11] Peckham H, de Gruijter NM, Raine C (2020). Male sex identified by global COVID-19 meta-analysis as a risk factor for death and ITU admission. Nat Commun.

[12] Zhou F, Yu T, Du R (2020). Clinical course and risk factors for mortality of adult inpatients with COVID-19 in Wuhan, China: a retrospective cohort study. Lancet.

[13] Anand A, Kumar R, Shalimar A (2020). Obesity and mortality in COVID-19: cause or association?. Gastroenterology.

[14] Bray I, Gibson A, White J (2020). Coronavirus disease 2019 mortality: a multivariate ecological analysis in relation to ethnicity, population density, obesity, deprivation and pollution. Public Health.

[15] Hu Z, Li S, Yang A (2021). Delayed hospital admission and high-dose corticosteroids potentially prolong SARS-CoV-2 RNA detection duration of patients with COVID-19. Eur J Clin Microbiol Infect Dis.

[16] Liang WH, Guan WJ, Li CC (2020). Clinical characteristics and outcomes of hospitalised patients with COVID-19 treated in Hubei (epicentre) and outside Hubei (non-epicentre): a nationwide analysis of China. Eur Respir J.

[17] Mackey K, Ayers CK, Kondo KK (2020). Racial and ethnic disparities in COVID-19-related infections, hospitalizations, and deaths: a systematic review. Ann Intern Med.

[18] Arrazola J, Masiello MM, Joshi S (2020). COVID-19 mortality among American Indian and Alaska native persons: 14 states, January–June 2020. MMWR Morb Mortal Wkly Rep.

[19] Cobre AF, Boger B, Fachi MM (2020). Risk factors associated with delay in diagnosis and mortality in patients with COVID-19 in the city of Rio de Janeiro, Brazil. Cien Saude Colet.

[20] Huang G, Gong T, Wang G (2020). Timely diagnosis and treatment shortens the time to resolution of coronavirus disease (COVID-19) pneumonia and lowers the highest and last CT scores from sequential chest CT. AJR Am J Roentgenol.

[21] Khonyongwa K, Taori SK, Soares A (2020). Incidence and outcomes of healthcare-associated COVID-19 infections: significance of delayed diagnosis and correlation with staff absence. J Hosp Infect.

[22] Li L, Sun W, Han M (2020). A study on the predictors of disease severity of COVID-19. Med Sci Monit.

[23] Tan L, Kang X, Ji X (2020). Validation of predictors of disease severity and outcomes in COVID-19 patients: a descriptive and retrospective study. Medicine (NY).

[24] Xu K, Zhou M, Yang D (2020). Application of ordinal logistic regression analysis to identify the determinants of illness severity of COVID-19 in China. Epidemiol Infect.

[25] Pfoh ER, Hariri EH, Misra-Hebert AD (2020). Late diagnosis of COVID-19 in patients admitted to the hospital. J Gen Intern Med.

[26] Leung K, Wu JT, Liu D (2020). First-wave COVID-19 transmissibility and severity in China outside Hubei after control measures, and second-wave scenario planning: a modelling impact assessment. Lancet.

[27] National Health Commission of the People's Republic of China. The latest situation of the new coronavirus pneumonia as of 24:00 on October 8. 2020. http://www.nhc.gov.cn/xcs/yqtb/202010/2fbce5a9836d4b09a89a0d85a2e05ac2.shtml (2020). accessed 22 Feb 2022

[28] National Health Commission of the People's Republic of China. COVID-19 included in the management of notifiable infectious diseases 2020. http://www.gov.cn/xinwen/2020-01/21/content_5471153.htm (2020). accessed 22 Feb 2022

[29] National Health Commission of the People’s Republic of China. diagnosis and treatment scheme of new coronavirus infected pneumonia (7th). 2020. http://www.nhc.gov.cn/xcs/zhengcwj/202003/46c9294a7dfe4cef80dc7f5912eb1989.shtml (2020). accessed 22 Feb 2022

[30] Franceschi C, Garagnani P, Parini P (2018). Inflammaging: a new immune-metabolic viewpoint for age-related diseases. Nat Rev Endocrinol.

[31] Panda A, Arjona A, Sapey E (2009). Human innate immunosenescence: causes and consequences for immunity in old age. Trends Immunol.

[32] Chen Y, Klein SL, Garibaldi BT (2021). Aging in COVID-19: vulnerability, immunity and intervention. Ageing Res Rev.

[33] Jergović M, Coplen CP, Uhrlaub JL (2021). Immune response to COVID-19 in older adults. J Heart Lung Transpl.

[34] Takahashi T, Ellingson MK, Wong P (2020). Sex differences in immune responses that underlie COVID-19 disease outcomes. Nature.

[35] Miao J, Li J, Wang F (2022). Characterization and evaluation of the leachability of bottom ash from a mobile emergency incinerator of COVID-19 medical waste: a case study in Huoshenshan Hospital, Wuhan, China. J Environ Manage.

[36] China Central Television. Makeshift hospitals start to accept patients in China's Wuhan. 2020. http://english.cctv.com/2020/02/06/ARTIxIHp9AN2fAStmOl5jYeE200206.shtml (2020). accessed 22 Feb 2022

[37] Ji Y, Ma Z, Peppelenbosch MP (2020). Potential association between COVID-19 mortality and health-care resource availability. Lancet Glob Health.

[38] Lee CCM, Thampi S, Lewin B (2020). Battling COVID-19: critical care and peri-operative healthcare resource management strategies in a tertiary academic medical centre in Singapore. Anaesthesia.

[39] Bedford J, Enria D, Giesecke J (2020). COVID-19: towards controlling of a pandemic. Lancet.

[40] Lonergan M, Chalmers JD (2020). Estimates of the ongoing need for social distancing and control measures post-"lockdown" from trajectories of COVID-19 cases and mortality. Eur Respir J.

[41] Nussbaumer-Streit B, Mayr V, Dobrescu AI (2020). Quarantine alone or in combination with other public health measures to control COVID-19: a rapid review. Cochrane Database Syst Rev.

[42] Pan A, Liu L, Wang C (2020). Association of public health Interventions with the epidemiology of the COVID-19 outbreak in Wuhan, China. JAMA.

[43] Siedner MJ, Harling G, Reynolds Z (2020). Social distancing to slow the US COVID-19 epidemic: longitudinal pretest-posttest comparison group study. PLoS Med.

