# Supplementary material for: Decline of onset-to-diagnosis interval and its impacts on clinical outcome of COVID-19 in China: a nation-wide observational study
Source: BMC Infect Dis. 2022 Aug 5;22:674. doi: 10.1186/s12879-022-07660-4 (PMC9356511; doi:10.1186/s12879-022-07660-4)
Supplement: Supplementary file 1 — Additional file 1: Fig S1. Geographical distributions of onset-to-diagnosis interval, severe rate (SR) and case fatality rate (CFR). Fig S2. Frequency distribution of onset-to-diagnosis interval of confirmed COVID-19 cases by regions and epidemic periods. Fig S3: The onset-to-diagnosis interval-related COVID-19 disease severe rate and case fatality rate stratified by regions. Fig S4. The odds ratio and attributable fraction of COVID-19 disease severe rate resulted from onset-to-diagnosis intervals in Wuhan and outside Wuhan. Fig S5. The odds ratio and attributable fraction of COVID-19 case fatality rate resulted from onset-to-diagnosis intervals in Wuhan and outside Wuhan. Table S1. The subgroup analysis on the onset-to-diagnosis interval of patients with COVID-19 for age, sex, geographic region and epidemic period by multivariate linear regression. Table S2. Odds ratio of onset-to-diagnosis interval for the risk of severe COVID-19 by multivariate logistic regression model. Table S3. Attributable fraction of onset-to-diagnosis interval for the risk of severe COVID-19. Table S4. Odds ratio of onset-to-diagnosis interval for the risk of fatal COVID-19 by multivariate logistic regression model. Table S5. Attributable fraction of onset-to-diagnosis interval for the risk of fatal COVID-19. Table S6. The predicted and actual case number of severe and death cases according to the different cutoff value of onset-to-diagnosis interval. [file 12879_2022_7660_MOESM1_ESM.docx]

**Supporting information captions**

| **Page** | **Item** |
| --- | --- |
| 2 | Figure S1: Geographical distributions of onset-to-diagnosis interval, severe rate (SR) and case fatality rate (CFR) |
| 3 | Figure S2: Frequency distribution of onset-to-diagnosis interval of confirmed COVID-19 cases by regions and epidemic periods. |
| 4 | Table S1: The subgroup analysis on the onset-to-diagnosis interval of patients with COVID-19 for age, sex, geographic region and epidemic period by multivariate linear regression |
| 5 | Figure S3: The onset-to-diagnosis interval-related COVID-19 disease severe rate and case fatality rate stratified by regions |
| 6 | Table S2: Odds ratio of onset-to-diagnosis interval for the risk of severe COVID-19 by multivariate logistic regression model |
| 7 | Table S3: Attributable fraction of onset-to-diagnosis interval for the risk of severe COVID-19 |
| 8 | Figure S4: The odds ratio and attributable fraction of COVID-19 disease severe rate resulted from onset-to-diagnosis intervals in Wuhan and outside Wuhan |
| 9 | Table S4: Odds ratio of onset-to-diagnosis interval for the risk of fatal COVID-19 by multivariate logistic regression model |
| 10 | Table S5: Attributable fraction of onset-to-diagnosis interval for the risk of fatal COVID-19 |
| 11 | Figure S5: The odds ratio and attributable fraction of COVID-19 case fatality rate resulted from onset-to-diagnosis intervals in Wuhan and outside Wuhan |
| 12 | Table S6: The predicted and actual case number of severe and death cases according to the different cutoff value of onset-to-diagnosis interval |

**Figure S1: Geographical distributions of onset-to-diagnosis interval, severe rate (SR) and case fatality rate (CFR).** (A) SR and onset-to-diagnosis interval in the whole study period; (B) SR and onset-to-diagnosis interval in Wuhan at different epidemic periods; (C) CFR and onset-to-diagnosis interval in the whole study period; (D) CFR and onset-to-diagnosis interval in Wuhan at different epidemic periods.


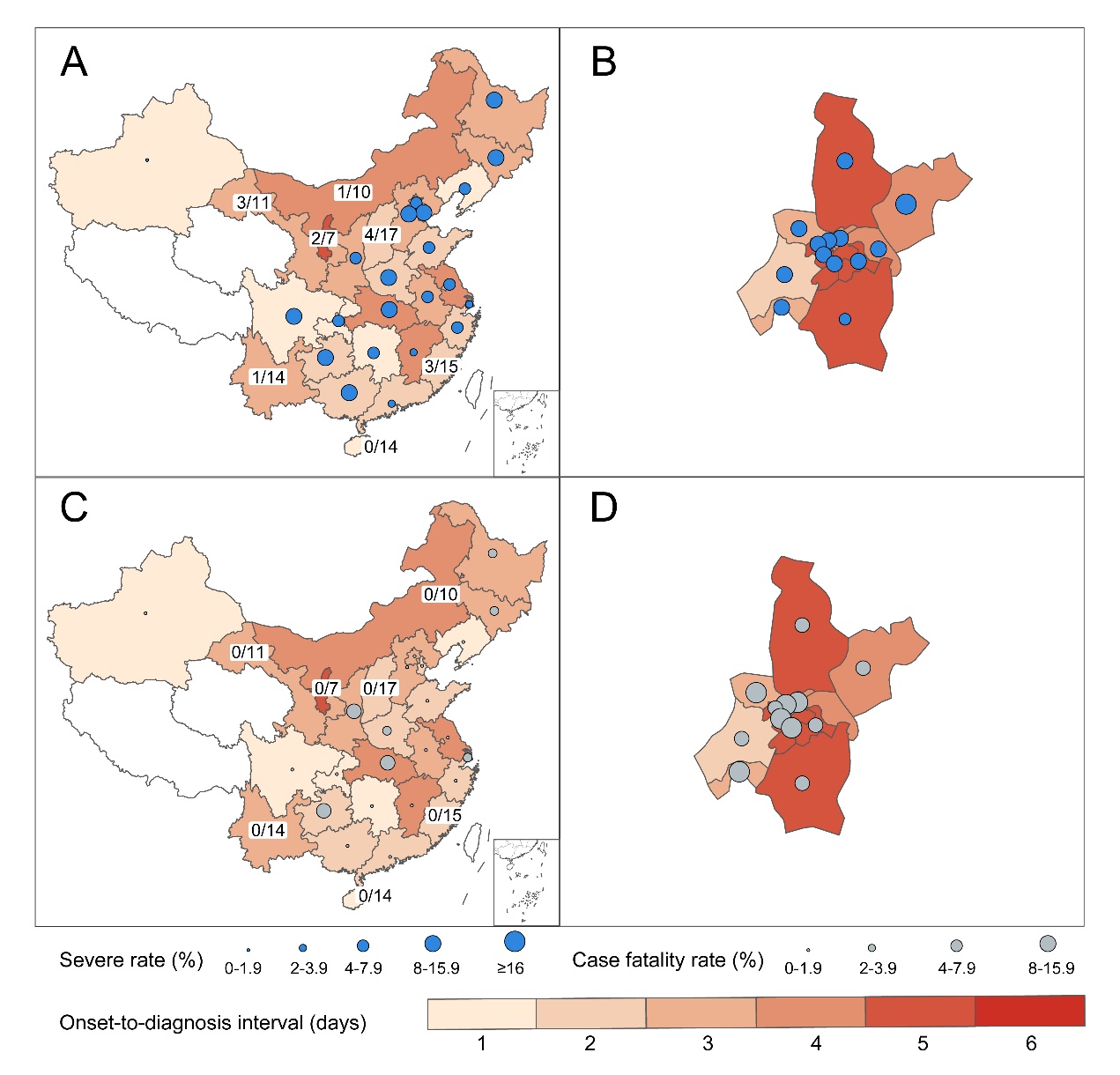


**Figure S2: Frequency distribution of onset-to-diagnosis interval of confirmed COVID-19 cases by regions and epidemic periods.** The median of onset-to-diagnosis interval was shown by purple dashed line in each panel.


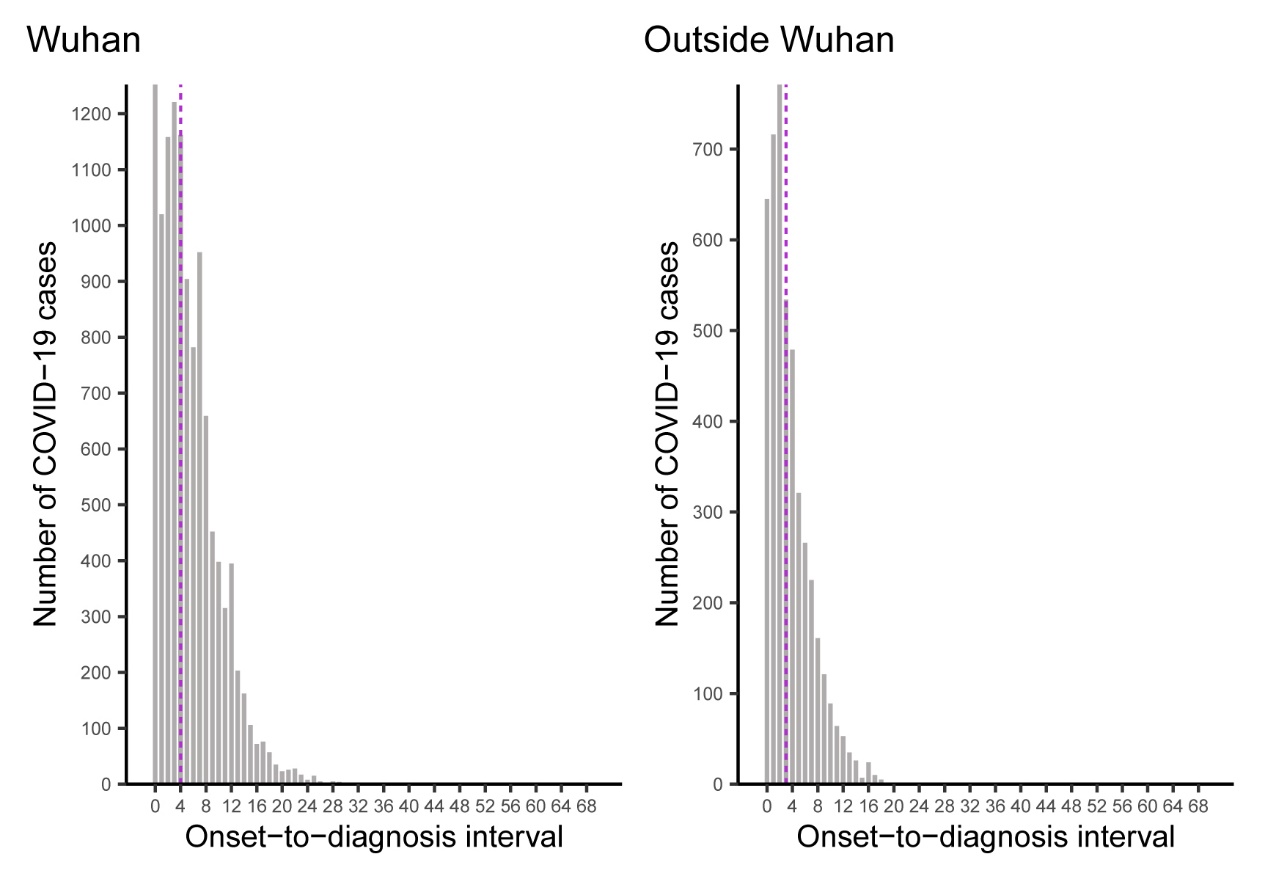


**Table S1.** **The subgroup analysis on the onset-to-diagnosis interval of patients with COVID-19 for age, sex, geographic region and epidemic period by** **multivariate linear regression model.**

| **Group** | **Onset-to-diagnosis interval^†^**  **Median (IQR)** | | **β (95% CI)** | **P-value** |
| --- | --- | --- | --- | --- |
| **All cases** |  |  | |  |
| **Age, years** |  |  | |  |
| 0‒59 | 4 (2-7) | Reference | | - |
| 60‒69 | 4 (2-7) | 0.243 (0.083, 0.402) | | 0.003 |
| ≥70 | 4 (2-7) | -0.01 (-0.179, 0.158) | | 0.904 |
| **Sex** |  |  | |  |
| Male | 4 (2-7) | Reference | | - |
| Female | 4 (2-7) | 0.328 (0.196, 0.461) | | <0.001 |
| **Region** |  |  | |  |
| Outside Wuhan | 3 (1-5) | Reference | | - |
| Wuhan | 4 (2-8) | 1.753 (1.605, 1.900) | | <0.001 |
| **Wuhan** |  |  | |  |
| **Age, years** |  |  | |  |
| 0‒59 | 4 (2-8) | Reference | | - |
| 60‒69 | 5 (2-8) | 0.161 (-0.038, 0.361) | | 0.113 |
| ≥70 | 4 (2-8) | -0.052 (-0.261, 0.157) | | 0.627 |
| **Sex** |  |  | |  |
| Male | 4 (2-7) | Reference | | - |
| Female | 5 (2-8) | 0.482 (0.315, 0.65) | | <0.001 |
| **Outside Wuhan** |  |  | |  |
| **Age, years** |  |  | |  |
| 0‒59 | 3 (1-5) | Reference | | - |
| 60‒69 | 3 (1-6) | 0.476 (0.231, 0.722) | | <0.001 |
| ≥70 | 3 (1-5) | 0.102 (-0.164, 0.367) | | 0.453 |
| **Sex** |  |  | |  |
| Male | 3 (1-5) | Reference | | - |
| Female | 3 (1-5) | -0.062 (-0.258, 0.133) | | 0.531 |

^†^The median (IQR) of onset-to-diagnosis interval was indicated.

The β>0 indicates the positive correlation with the onset-to-diagnosis interval; the β<0 indicates the negative correlation with the onset-to-diagnosis interval.

**Figure S3:** **The onset-to-diagnosis interval-related COVID-19 disease severe rate and case fatality rate stratified by regions.**

(A) severe rate by sex in Wuhan; (B) severe rate by age in Wuhan; (C) severe rate by sex outside Wuhan; (D) severe rate by age outside Wuhan; (E) case fatality rate by sex in Wuhan; (F) case fatality rate by age in Wuhan; (G) case fatality rate by sex outside Wuhan; (H) case fatality rate by age outside Wuhan.


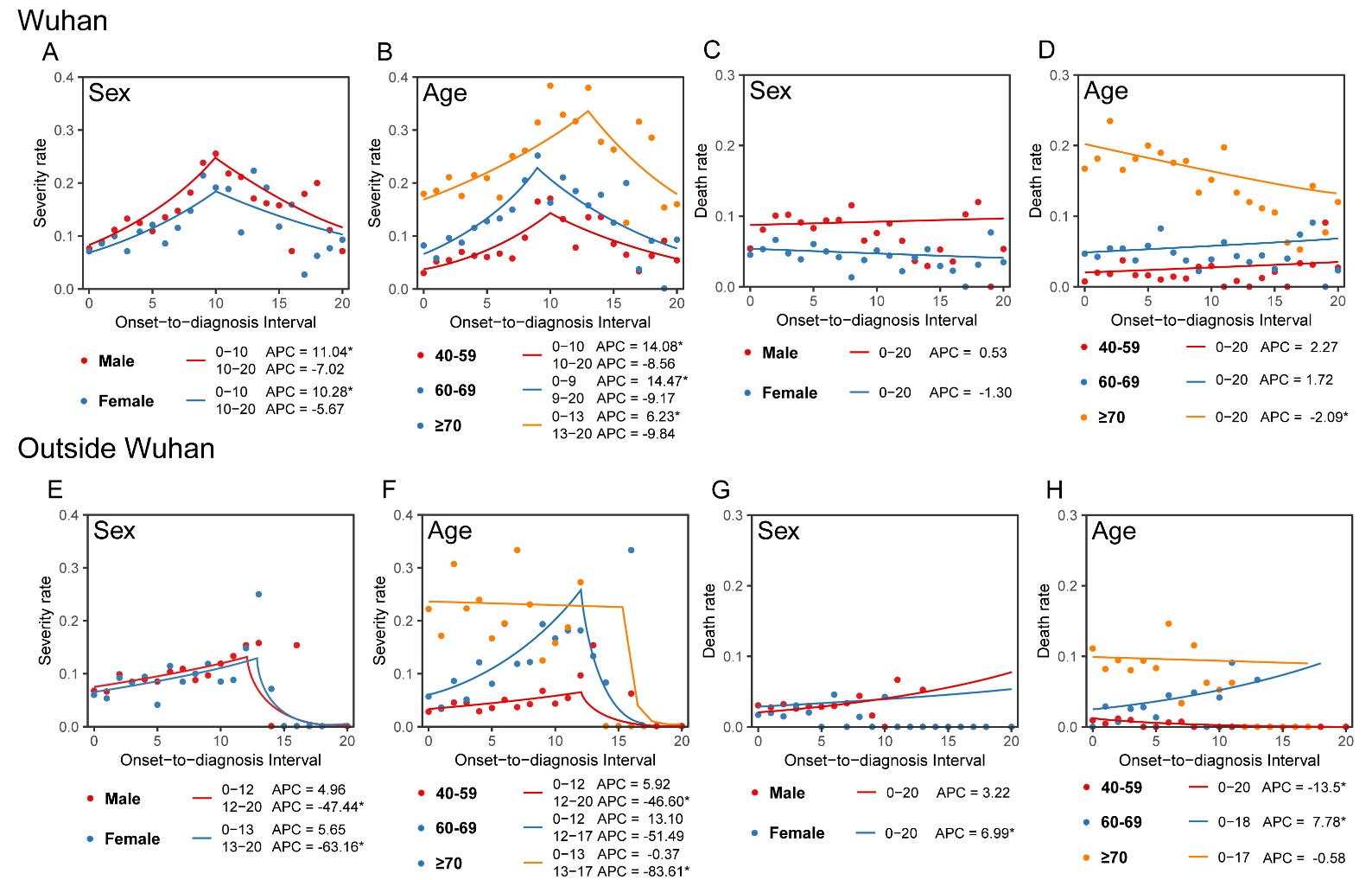


**Table S2: Odds ratio of onset-to-diagnosis interval for the risk of severe COVID-19 by multivariate logistic regression model**

| **Group** | **0-1 Days** | **2-3 Days** | **4-5 Days** | **6-7 Days** | **8-9 Days** | **10-11 Days** | **12-13 Days** | **14-15 Days** | **16-17 Days** | **18-19 Days** |
| --- | --- | --- | --- | --- | --- | --- | --- | --- | --- | --- |
| **All cases*** | Reference | 1.34 (1.13, 1.59) | 1.43 (1.20, 1.71) | 1.65 (1.37, 1.98) | 2.61 (2.15, 3.17) | 2.95 (2.37, 3.66) | 2.71 (2.11, 3.48) | 2.25 (1.58, 3.22) | 1.38 (0.83, 2.29) | 1.28 (0.64, 2.56) |
| **Sex^#^** |  |  |  |  |  |  |  |  |  |  |
| Female | Reference | 1.20 (0.94, 1.54) | 1.49 (1.16, 1.92) | 1.52 (1.17, 1.98) | 2.64 (2.01, 3.45) | 2.77 (2.03, 3.78) | 2.72 (1.95, 3.81) | 2.47 (1.56, 3.93) | 1.13 (0.53, 2.42) | 0.86 (0.30, 2.47) |
| Male | Reference | 1.47 (1.17, 1.86) | 1.37 (1.07, 1.76) | 1.78 (1.37, 2.31) | 2.58 (1.96, 3.40) | 3.11 (2.29, 4.22) | 2.72 (1.86, 3.96) | 1.99 (1.13, 3.49) | 1.65 (0.83, 3.27) | 1.89 (0.74, 4.83) |
| **Age^†^** |  |  |  |  |  |  |  |  |  |  |
| 40-59 | Reference | 1.57 (1.16, 2.13) | 1.47 (1.06, 2.04) | 1.67 (1.19, 2.34) | 3.23 (2.31, 4.50) | 4.08 (2.80, 5.93) | 3.00 (1.97, 4.57) | 3.13 (1.74, 5.61) | 1.34 (0.48, 3.76) | 1.77 (0.53, 5.84) |
| 60-69 | Reference | 1.36 (0.95, 1.94) | 1.93 (1.36, 2.75) | 2.48 (1.74, 3.56) | 3.95 (2.73, 5.72) | 3.20 (2.11, 4.86) | 3.04 (1.88, 4.91) | 2.46 (1.31, 4.62) | 2.11 (0.91, 4.89) | 0.70 (0.09, 5.30) |
| ≥70 | Reference | 1.19 (0.93, 1.53) | 1.18 (0.91, 1.54) | 1.26 (0.95, 1.67) | 1.61 (1.17, 2.20) | 2.13 (1.52, 3.01) | 2.29 (1.52, 3.45) | 1.54 (0.83, 2.84) | 1.03 (0.46, 2.27) | 1.26 (0.50, 3.18) |
| **Region^‡^** |  |  |  |  |  |  |  |  |  |  |
| Outside Wuhan | Reference | 1.51 (1.12, 2.05) | 1.30 (0.92, 1.85) | 1.88 (1.28, 2.75) | 1.77 (1.11, 2.82) | 1.57 (0.87, 2.82) | 3.06 (1.63, 5.78) | 0.55 (0.07, 4.22) | 0.89 (0.20, 3.93) | 0.00  (0.00, ∞) |
| Wuhan | Reference | 1.27 (1.03, 1.56) | 1.45 (1.18, 1.79) | 1.57 (1.27, 1.94) | 2.73 (2.19, 3.39) | 3.13 (2.46, 3.99) | 2.56 (1.93, 3.38) | 2.33 (1.61, 3.38) | 1.43 (0.83, 2.45) | 1.27 (0.64, 2.55) |

^*^Adjusted the variables of age, sex and region.

^#^Adjusted the variables of age and region.

**^†^**Adjusted the variables of sex and region.

**^‡^**Adjusted the variables of age and sex.

**Table S3: Attributable fraction of onset-to-diagnosis interval for the risk of severe COVID-19**

| **Group** | **0-1 Days** | **2-3 Days** | **4-5 Days** | **6-7 Days** | **8-9 Days** | **10-11 Days** | **12-13 Days** | **14-15 Days** | **16-17 Days** | **18-19 Days** |
| --- | --- | --- | --- | --- | --- | --- | --- | --- | --- | --- |
| **All cases*** | Reference | 14.6 (4.9, 24.4) | 16.1 (6.7, 25.5) | 19.4 (10.7, 28.1) | 29.1 (22.2, 36.1) | 25.6 (18.4, 32.8) | 18.6 (10.5, 26.8) | 7.9 (-2.1, 17.9) | 1.8 (-9.4, 13.1) | 0.8 (-10.6, 12.3) |
| **Sex^#^** |  |  |  |  |  |  |  |  |  |  |
| Female | Reference | 9.1 (-60, 24.2) | 17.3 (4.4, 30.3) | 16.8 (3.8, 29.8) | 29.5 (19.8, 39.3) | 23.7 (13.2, 34.3) | 20.7 (9.5, 31.9) | 10.1 (-3.4, 23.7) | 0.7 (-15.7, 170) | -0.5 (-17.1, 16.2) |
| Male | Reference | 19.6 (6.8, 32.4) | 14.7 (1.0, 28.3) | 21.8 (10.1, 33.5) | 28.7 (18.8, 38.6) | 27.4 (17.5, 37.2) | 16.5 (4.5, 28.5) | 5.6 (-9.1, 20.4) | 3.0 (-12.5, 18.5) | 2.0 (-13.7, 17.8) |
| **Age^†^** |  |  |  |  |  |  |  |  |  |  |
| 40-59 | Reference | 21.7 (5.5, 37.9) | 16.1 (-1.4, 33.7) | 19.2 (2.6, 35.7) | 35.4 (23.8, 47.1) | 31.4 (19.3, 43.5) | 22.4 (7.8, 37.1) | 11.6 (-6.3, 29.4) | 1.3 (-20.7, 23.3) | 1.7 (-20.1, 23.5) |
| 60-69 | Reference | 15.3 (-5.6, 36.3) | 29.8 (13.6, 460) | 35.9 (21.8, 50.1) | 43.8 (32.1, 55.5) | 31.7 (17.5, 45.9) | 23.7 (7.5, 39.9) | 12.0 (-8.2, 32.3) | 5.9 (-16.9, 28.8) | -0.8 (-26.8, 25.3) |
| ≥70 | Reference | 9.1 (-5.9, 24.1) | 7.8 (-7.2, 22.8) | 9.0 (-5.3, 23.4) | 14.2 (1.4, 26.9) | 17.8 (6.3, 29.3) | 13.7 (1.7, 25.8) | 3.4 (-11, 17.8) | 0.1 (-15.1, 15.4) | 0.9 (-14.1, 15.9) |
| **Region^‡^** |  |  |  |  |  |  |  |  |  |  |
| Outside Wuhan | Reference | 19.9 (3.9, 35.9) | 10.1 (-80, 28.3) | 17.7 (2.4, 330) | 10.9 (-6.2, 27.9) | 5.8 (-12.9, 24.6) | 10.2 (-6.7, 27.2) | -10 (-22.1, 20.2) | -0.3 (-21.2, 20.6) | 0 (-20.7, 20.7) |
| Wuhan | Reference | 12.2 (-0.1, 24.5) | 17.7 (6.6, 28.9) | 19.3 (8.6, 30.0) | 33.9 (26.1, 41.8) | 31.1 (23.1, 390) | 21.1 (11.6, 30.6) | 110 (-0.4, 22.3) | 2.6 (-10.8, 160) | 1.1 (-12.6, 14.9) |

**Figure S4: The odds ratio and attributable fraction of COVID-19 disease severe rate resulted from onset-to-diagnosis intervals in Wuhan and outside Wuhan.** (A) by sex in Wuhan; (B) by sex outside Wuhan; (C) by age in Wuhan; (D) by age outside Wuhan. The points and lines represent ORs and their 95% CIs. The bars represent the AFs and their significance of differences by asterisk (*, P<0.05; **, P<0.01).

**
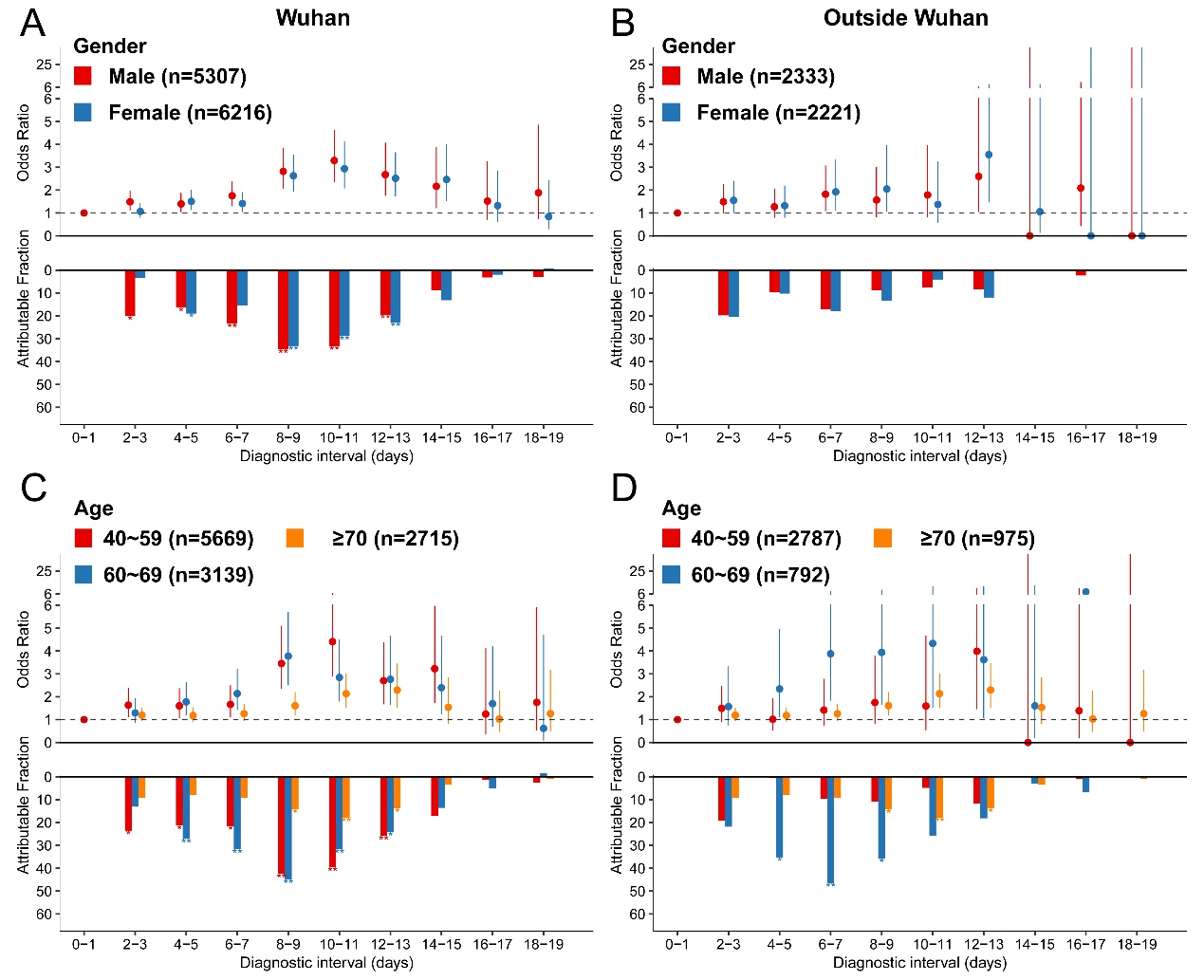
**

**Table S4: Odds ratio of onset-to-diagnosis interval for the risk of fatal COVID-19 by multivariate logistic regression model**

| **Group** | **0-1 Days** | **2-3 Days** | **4-5 Days** | **6-7 Days** | **8-9 Days** | **10-11 Days** | **12-13 Days** | **14-15 Days** | **16-17 Days** | **18-19 Days** |
| --- | --- | --- | --- | --- | --- | --- | --- | --- | --- | --- |
| **All cases*** | Reference | 1.27 (1.03, 1.58) | 1.11 (0.88, 1.41) | 1.28 (0.99, 1.63) | 1.01 (0.74, 1.37) | 1.12 (0.80, 1.57) | 0.78 (0.49, 1.23) | 0.80 (0.42, 1.53) | 0.63 (0.27, 1.46) | 1.17 (0.49, 2.82) |
| **Sex^#^** |  |  |  |  |  |  |  |  |  |  |
| Female | Reference | 1.12 (0.80, 1.57) | 0.95 (0.66, 1.38) | 1.08 (0.74, 1.57) | 0.51 (0.29, 0.90) | 1.08 (0.64, 1.83) | 0.78 (0.40, 1.52) | 1.03 (0.45, 2.33) | 0.23 (0.03, 1.70) | 1.19 (0.35, 4.11) |
| Male | Reference | 1.39 (1.05, 1.85) | 1.24 (0.92, 1.69) | 1.45 (1.05, 2.01) | 1.45 (1.00, 2.09) | 1.16 (0.75, 1.82) | 0.80 (0.42, 1.50) | 0.58 (0.21, 1.65) | 0.97 (0.37, 2.54) | 1.17 (0.34, 4.07) |
| **Age^†^** |  |  |  |  |  |  |  |  |  |  |
| 40-59 | Reference | 2.03 (1.20, 3.46) | 1.06 (0.55, 2.05) | 1.16 (0.58, 2.33) | 1.40 (0.65, 2.99) | 1.30 (0.49, 3.47) | 0.63 (0.15, 2.73) | 1.43 (0.33, 6.17) | 1.18 (0.16, 8.91) | 5.12 (1.14, 22.91) |
| 60-69 | Reference | 1.18 (0.73, 1.90) | 1.12 (0.67, 1.86) | 1.68 (1.02, 2.77) | 0.82 (0.41, 1.66) | 1.39 (0.72, 2.70) | 1.12 (0.49, 2.60) | 0.87 (0.26, 2.91) | 1.55 (0.46, 5.24) | 1.28 (0.17, 9.87) |
| ≥70 | Reference | 1.14 (0.87, 1.50) | 1.13 (0.84, 1.50) | 1.17 (0.86, 1.60) | 1.00 (0.69, 1.46) | 1.01 (0.66, 1.56) | 0.70 (0.39, 1.27) | 0.67 (0.28, 1.59) | 0.28 (0.07, 1.19) | 0.72 (0.21, 2.42) |
| **Region^‡^** |  |  |  |  |  |  |  |  |  |  |
| Outside Wuhan | Reference | 1.05 (0.64, 1.74) | 0.83 (0.45, 1.53) | 1.30 (0.68, 2.51) | 0.94 (0.38, 2.31) | 0.90 (0.31, 2.65) | 0.40 (0.05, 3.00) | ‒ | ‒ | ‒ |
| Wuhan | Reference | 1.31 (1.03, 1.67) | 1.09 (0.84, 1.41) | 1.15 (0.87, 1.50) | 0.90 (0.65, 1.25) | 1.02 (0.71, 1.46) | 0.71 (0.44, 1.15) | 0.72 (0.38, 1.37) | 0.61 (0.26, 1.44) | 0.97 (0.40, 2.35) |

^*^Adjusted the variables of age, sex and region.

^#^Adjusted the variables of age and region.

**^†^**Adjusted the variables of sex and region.

**^‡^**Adjusted the variables of age and sex.

**Table S5: Attributable fraction of onset-to-diagnosis interval for the risk of fatal COVID-19**

| **Group** | **0-1 Days** | **2-3 Days** | **4-5 Days** | **6-7 Days** | **8-9 Days** | **10-11 Days** | **12-13 Days** | **14-15 Days** | **16-17 Days** | **18-19 Days** |
| --- | --- | --- | --- | --- | --- | --- | --- | --- | --- | --- |
| **All cases*** | Reference | 12.4 (-0.6, 25.4) | 5.1 (-9.5, 19.6) | 9.6 (-3.7, 22.9) | 0.3 (-15.1, 15.7) | 2.5 (-12.1, 17.2) | -3.6 (-19.8, 12.7) | -1.6 (-17.2, 14.1) | -2.1 (-17.9, 13.6) | 0.5 (-14.4, 15.5) |
| **Sex^#^** |  |  |  |  |  |  |  |  |  |  |
| Female | Reference | 5.7 (-16, 27.5) | -2.2 (-26.5, 22.2) | 3.0 (-19.5, 25.4) | -17 (-47.4, 13.5) | 1.7 (-20.8, 24.2) | -3.8 (-28.6, 210) | 0.2 (-22.6, 23.1) | -4.7 (-29.7, 20.4) | 0.7 (-21.9, 23.3) |
| Male | Reference | 16.9 (0.8, 33.1) | 10.4 (-7.6, 28.3) | 14.4 (-1.9, 30.7) | 11.2 (-5.7, 28.2) | 3.5 (-15.7, 22.7) | -30.0 (-24.3, 18.4) | -30.0 (-24.3, 18.3) | -0.1 (-20.3, 20.0) | 0.5 (-19.4, 20.4) |
| **Age^†^** |  |  |  |  |  |  |  |  |  |  |
| 40-59 | Reference | 33.6 (9.0, 58.2) | 2.6 (-38.5, 43.6) | 5.2 (-34.1, 44.6) | 9.2 (-27.1, 45.4) | 4.4 (-34.8, 43.6) | -5.1 (-52.1, 41.9) | 2.6 (-37.8, 43.1) | 0.7 (-41.3, 42.7) | 7.0 (-29.9, 43.9) |
| 60-69 | Reference | 8.3 (-22.8, 39.4) | 5.4 (-27, 37.8) | 21.2 (-3.3, 45.8) | -5.6 (-44, 32.8) | 8.3 (-21.8, 38.4) | 2.0 (-31.5, 35.6) | -1.3 (-36.9, 34.3) | 3.1 (-29.5, 35.7) | 0.7 (-33.5, 34.8) |
| ≥70 | Reference | 7.0 (-10.1, 24.2) | 5.6 (-11.7, 22.9) | 6.3 (-10.5, 23.1) | 0.0 (-18.2, 18.2) | 0.3 (-17.6, 18.2) | -4.8 (-24, 14.5) | -2.6 (-21, 15.8) | -4.5 (-23.5, 14.5) | -10 (-18.8, 16.7) |
| **Region^‡^** |  |  |  |  |  |  |  |  |  |  |
| Outside Wuhan | Reference | 2.6 (-31.1, 36.3) | -7.1 (-46.1, 31.9) | 7.1 (-23.4, 37.5) | -1.1 (-36.3, 34.1) | -1.2 (-36.4, 340) | -4.6 (-42, 32.9) | ‒ | ‒ | ‒ |
| Wuhan | Reference | 13.9 (-0.3, 28.2) | 4.2 (-12.4, 20.8) | 6.0 (-10, 21.9) | -3.5 (-21.8, 14.9) | 0.5 (-16.6, 17.5) | -5.8 (-24.6, 13.1) | -3.1 (-20.9, 14.8) | -2.8 (-20.6, 14.9) | -0.1 (-17.0, 16.7) |

**Figure S5: The odds ratio and attributable fraction of COVID-19 case fatality rate resulted from onset-to-diagnosis intervals in Wuhan and outside Wuhan.** (A) by sex in Wuhan; (B) by sex outside Wuhan; (C) by age in Wuhan; (D) by age outside Wuhan. The points and lines represent ORs and their 95% CIs. The bars represent the AFs and their significance of differences by asterisk (*, P<0.05; **, P<0.01).

**
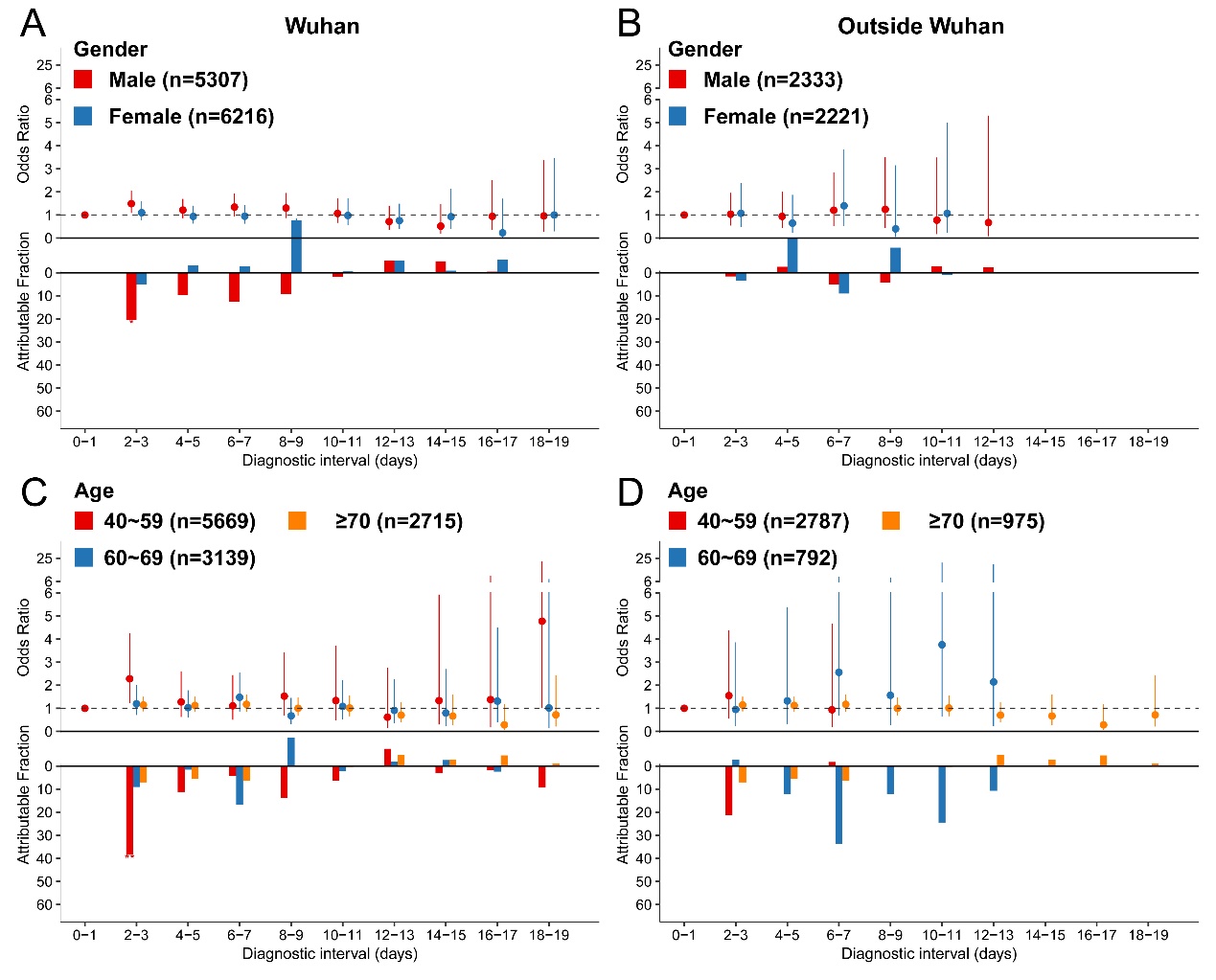
**

**Table S6: The predicted number of severe and death cases according to the different cutoff value of onset-to-diagnosis interval, compared to the actual numbers reported.**

| **Cutoff value** | **Predicted severe cases (95% CI)** | **Reduced severe cases (95% CI)** | **Predicted death cases (95% CI)** | **Reduced death cases (95% CI)** |
| --- | --- | --- | --- | --- |
| 0 | 1236 (683, 2047) | 556 (-255, 1109) | 764 (405, 1422) | 73 (-585, 432) |
| 1 | 1233 (823, 1773) | 559 (19, 969) | 798 (507, 1234) | 39 (-397, 330) |
| 2 | 1371 (1013, 1809) | 421 (-17, 779) | 881 (631, 1225) | -44 (-388, 206) |
| 3 | 1398 (1082, 1774) | 394 (18, 710) | 891 (666, 1185) | -54 (-348, 171) |
| 4 | 1456 (1163, 1797) | 336 (-5, 629) | 873 (672, 1130) | -36 (-293, 165) |
| 5 | 1480 (1203, 1800) | 312 (-8, 589) | 876 (687, 1114) | -39 (-277, 150) |
| 6 | 1506 (1238, 1812) | 286 (-20, 554) | 888 (705, 1115) | -51 (-278, 132) |
| 7 | 1550 (1291, 1843) | 242 (-51, 501) | 881 (708, 1095) | -44 (-258, 129) |
| 8 | 1603 (1348, 1891) | 189 (-99, 444) | 875 (708, 1081) | -38 (-244, 129) |
| 9 | 1672 (1416, 1957) | 120 (-165, 376) | 865 (702, 1065) | -28 (-228, 135) |
| 10 | 1720 (1466, 2003) | 72 (-211, 326) | 862 (701, 1058) | -25 (-221, 136) |
| 11 | 1751 (1498, 2032) | 41 (-240, 294) | 861 (703, 1054) | -24 (-217, 134) |
| 12 | 1770 (1520, 2049) | 22 (-257, 272) | 855 (699, 1044) | -18 (-207, 138) |
| 13 | 1791 (1541, 2068) | 1 (-276, 251) | 849 (695, 1036) | -12 (-199, 142) |
| 14 | 1800 (1551, 2076) | -8 (-284, 241) | 845 (693, 1031) | -8 (-194, 144) |
| 15 | 1801 (1553, 2076) | -9 (-284, 239) | 844 (692, 1029) | -7 (-192, 145) |

Note: The actual numbers of severe cases and deaths are 1792 and 837, respectively.
